# Supplementary material for: Polyketide synthase gene clusters in the ericoid mycorrhizal fungus Oidiodendron maius: genome-wide prediction, expression profiling, and functional characterization of knockout mutants
Source: Front Plant Sci. 2026 Sep 16;17:1924430. doi: 10.3389/fpls.2026.1924430 (PMC13623903; doi:10.3389/fpls.2026.1924430)
Supplement: Supplementary file 1 [file SupplementaryFile1.zip › Supplementary Materials/Supplementary Folder 1.zip!/Oidma1_AssemblyScaffolds_Repeatmasked/knownclusterblast/cluster55/jgi.p_Oidma1_173677_mibig_hits.html]

| MIBiG Protein | Description | MIBiG Cluster | MiBiG Product | % ID | % Coverage | BLAST Score | E-value |
| --- | --- | --- | --- | --- | --- | --- | --- |
| AGO59041.1 | PtaB | BGC0000121 | Pestheic\_acid\_biosynthetic\_gene\_cluster | 69 | 99.3670886076 | 453 | 2e-127 |
| EAL89338.1 | metallo-beta-lactamase\_domain\_protein,\_putative | BGC0001403 | Trypacidin\_biosynthetic\_gene\_cluster | 66 | 104.746835443 | 449 | 2.9e-126 |
| ADM34149.1 | metallo-beta-lactamase\_domain\_protein | BGC0001084 | Notoamide\_/\_stephacidin\_biosynthetic\_gene\_cluster | 67 | 99.3670886076 | 427 | 1.2e-119 |
| CBF90099.1 | metallo-beta-lactamase\_domain\_protein,\_putative\_(AFU\_orthologue;\_AFUA\_4G14570) | BGC0001271 | Emericellin\_biosynthetic\_gene\_cluster | 63 | 98.417721519 | 413 | 1.8e-115 |
| AEN83888.1 | AdaB | BGC0000156 | TAN-1612\_biosynthetic\_gene\_cluster | 51 | 94.3037974684 | 316 | 5e-86 |
| EAL84396.1 | metallo-beta-lactamase\_domain\_protein | BGC0001118 | Endocrocin\_biosynthetic\_gene\_cluster | 50 | 95.253164557 | 307 | 2.3e-83 |
| CBF70385.1 | metallo-beta-lactamase\_domain\_protein\_(AFU\_orthologue;\_AFUA\_7G00120) | BGC0000684 | Asperthecin\_biosynthetic\_gene\_cluster | 50 | 93.3544303797 | 296 | 3.1e-80 |
| EGD99351.1 | metallo-beta-lactamase | BGC0001144 | Neosartoricin\_biosynthetic\_gene\_cluster | 47 | 99.6835443038 | 277 | 2e-74 |
| ADI24932.1 | VrtG | BGC0000168 | Viridicatumtoxin\_biosynthetic\_gene\_cluster | 48 | 93.9873417722 | 274 | 1.3e-73 |
| CBF79145.1 | conserved\_hypothetical\_protein | BGC0000013 | Alternariol\_biosynthetic\_gene\_cluster | 53 | 70.5696202532 | 250 | 2.6e-66 |
